# How to Study the Effects of Dietary Lipids on the Small Intestinal Microbiome? Methodological Design and Evaluation of the Human HealThy fAt, haPpy mIcRobiome (TAPIR) Proof-of-Concept Study

**DOI:** 10.1016/j.cdnut.2025.104564

**Published:** 2025-02-05

**Authors:** Lonneke JanssenDuijghuijsen, Karen Fransen, Ruolei Deng, Corine Perenboom, Nicole de Wit, Guido Hooiveld, Mara van Trijp

**Affiliations:** 1 Wageningen Food and Biobased Research, Wageningen University & Research, Wageningen, the Netherlands; 2 Division of Human Nutrition and Health, Wageningen University & Research, Wageningen University, Wageningen, the Netherlands

**Keywords:** small intestinal microbiota, proof-of-concept human study, intestinal catheter, small intestine aspiration capsule, metabolites, mild-ketogenic diet, lipidomics, lipids, plant-based diet

## Abstract

**Background:**

Emerging evidence highlights the importance of the small intestinal microbiota in digestion and metabolism, underscoring the challenging need for human studies beyond fecal analyses.

**Objective:**

The TAPIR (acronym of “healthy fat, happy microbiome”) proof-of-concept study was primarily designed to confirm the interaction between the small intestinal microbiota and dietary lipids in healthy adults with a challenge test. We also aimed to assess the impact of a plant-based mild-ketogenic preconditioning diet on microbiome composition and function. Here, we comprehensively describe our extensive study protocol and evaluate the study execution.

**Methods:**

Participants consumed an 8-day preconditioning diet, followed by a high-fat shake challenge test on day 9. During this test, fasting and postprandial small intestinal aspirates were collected every 20 min via a naso-intestinal catheter, and blood samples were collected hourly. Participants ingested small intestine aspiration capsules before (day 0), on day 6 of the preconditioning diet, and during the challenge test. Dietary compliance, capsule retrieval, sample collection, stool pattern, and gastrointestinal complaints were monitored to evaluate study execution.

**Results:**

Twenty adults with a mean age of 48 y (19–88 y) and a mean body mass index (BMI) of 24.3 kg/m^2^ (19.5–30 kg/m^2^) consumed a preconditioning diet with a 96% compliance. There were no significant changes in gastrointestinal complaints and stool patterns during the study. Mean aspiration capsule retrieval rate was 94.7%, with mean sample weights per timepoint between 84.2 and 95.4 mg and median transit times between 32.8 and 49.3 h. The average success rate of aspirate collection by catheter was 49%, varying significantly between time points.

**Conclusion:**

The dietary intervention was successful and well-tolerated. We sampled in the small intestine with capsules and catheters, each with its own (dis)advantages. The comprehensive description and evaluation of our study execution offer practical insights supporting future study designs in food-microbe interactions in the small intestine.

The trial is registered at clinicaltrials.gov as NCT06064266.

## Introduction

The significance of measuring the human small intestinal microbiota has become increasingly evident in recent years [[1], [2], [3]]. Although current research on the small intestinal microbiota in healthy adults without gastrointestinal (GI) disorders remains limited [4], emerging evidence suggests its critical role in food digestion, metabolism, and absorption [5]. However, these findings on the relation between diet and the small intestinal microbiota primarily stem from animal studies and extrapolations from human fecal microbiome analyses, necessitating further investigation in human subjects. The major challenge in designing human intervention studies aimed at the small intestinal microbiota is the difficulty in obtaining local samples that accurately reflect the in-situ situation. Therefore, there is very limited knowledge about the interaction between diet and the small intestinal microbiota. For several decades, highly invasive intestinal catheters have been used to study the small intestinal microbiota in both healthy individuals and patients [6]. However, recent advancements in sampling techniques and novel study designs have opened new avenues to study the intricate relationship between diet and the human small intestinal microbiota. The use of innovative, less invasive aspiration capsules will expectedly further expand this research field [7]. These aspiration capsules potentially offer a new way to investigate how dietary components interact with the human small intestinal microbiota and their subsequent impact on human health, an area that is currently underexplored [7]. They also have great potential to further characterize the small intestinal microbiota community in large population cohorts or to investigate the effects of longer-term (nutritional) interventions on the small intestinal microbiota. These innovations have already yielded valuable insights into the small intestinal microbiome, metabolome, and proteome [4], underscoring the importance of integrating dietary data and microbial data to uncover novel diet-microbiome interactions [5,8]. Shalon et al. [4] study, however, lacked a controlled dietary intervention, relying solely on self-reported dietary intake. The capsules they used furthermore did not contain a stabilizing reagent, therefore, the samples are less likely to reliably reflect the *in situ* status. Moreover, to study food-microbe interactions in the small intestine over time, capsules are less suitable, because multiple capsules would be required to capture the *in vivo* dynamics over a period of several hours. Recent studies using catheters have demonstrated rapid changes within a day in the relative composition of human small intestinal microbiota following consumption of prebiotics [9] or synbiotics [10]. This demonstrated the importance of postprandial intestinal sampling in tracking acute responses of the small intestinal microbiota community to food compounds. To this end, intestinal catheters, despite their high burden on study participants, remain the gold standard for sampling in the small intestine over time. By comprehensively describing our study protocol and evaluating the study execution, including sampling procedures and the dietary intervention, we aimed to contribute to the growing body of literature on the interaction between diet and the small intestinal microbiota. Additionally, we hope to serve as a practical guide and reference for researchers designing and executing studies on food-microbe interactions in the small intestine. Gut health and microbiota research have primarily focused on how changes in digestible carbohydrates and, to a lesser extent, protein intake affect the microbiome and, consequently, health outcomes. Dietary lipids have received little attention in this respect, and their impact in the colon was always assumed to be limited due to highly efficient lipid digestion and absorption in humans, resulting in a near-to-complete absorption of fatty acids in the small intestine. Recent studies, however, suggest a role for the small intestinal microbiota in lipid digestion, metabolism, and absorption, potentially influencing individual responses to dietary lipids [5]. Recent discoveries also reveal that dietary lipid energy % (EN%) and origin (plant-based compared with animal-based) can exert a profound effect, within a remarkably short timeframe, on the composition and metabolic activity of the small intestine in preclinical animal models [11,12] and the fecal microbiota of human subjects [13]. Although previous research has explored the effects of dietary intake on duodenum microbial composition in healthy and obese individuals [14], to our knowledge, no study has specifically investigated the direct interaction between the small intestinal microbiota and dietary lipids *in vivo* in humans, with a focus on the analysis of microbial-derived metabolites in the postprandial state. The significance of the effects on human health remains largely unexplored due to the predominant focus on fecal-oriented microbiome analyses, which may fail to capture critical local effects occurring within the small intestine [11]. Combining different sampling techniques may offer the opportunity to study lipid digestion kinetics and the interaction with the small intestinal microbiota in detail.

For certain dietary lipids there is available literature on their potential for microbial transformation, primarily studied in animal models. For example, linoleic acid (LA) can be transformed by the microbiome into metabolites such as 0-Hydroxy-cis-12-octadecenoic acid and conjugated linoleic acid (CLA), both of which have potential health effects [[15], [16], [17], [18]]. Additionally, microbial fermentation of plant sterols typically results in the production of metabolites like ethyl-coprostanol and ethyl-coprostanone [8]. However, many microbiome-derived metabolites remain inadequately characterized, with their biological functions yet to be fully elucidated.

The TAPIR (acronym of “healthy fat, happy microbiome”) proof-of-concept study was primarily designed to confirm the interaction between the small intestinal microbiota and dietary lipids in healthy human adults. By employing small intestinal sampling via both naso-intestinal catheters (the gold standard) and novel, less invasive aspiration capsules, we intended to detect and confirm microbiota-derived lipid metabolites in the small intestine upon exposure to a high-fat challenge. Feces and blood were also collected to compare microbiota-derived lipid metabolites in various biological samples. An 8-d plant-based mild-ketogenic preconditioning diet that was integrated into the study design provided the opportunity to additionally explore the impact of a high-fat diet on microbiome composition and functional capacity in the small intestine and feces. In this publication, we thoroughly describe and evaluate our study design and execution, aiming to enhance the interpretability of results that will be obtained by future analyses and offer practical insights that support the set-up, execution, and reproducibility of future studies focusing on the interaction between diet and the human small intestinal microbiota.

## Methods

The TAPIR proof-of-concept study was performed between September and December 2023 in the Netherlands. Ethical approval was obtained from the Medical Ethical Committee Oost-Nederland (NL81345.081.23). The trial is registered at clinicaltrials.gov (NCT06064266) and conducted according to the declaration of Helsinki. Written informed consent was obtained from each participant prior to inclusion in the study.

### Study population

Generally healthy adult males and females with a BMI ranging from 18.5 to 30 kg/m^2^ were eligible for inclusion, with no upper age limit. Exclusion criteria included individuals with a history of medical or surgical events that could jeopardize their participation or influence study outcomes. Additionally, exclusion criteria included the use of antibiotics within 3 mo before study start or planned during the study, the use of medication affecting the GI motor function in the week before study start, and the use of pro- and prebiotic supplements within 4 wk before study start. Other exclusion criteria were individuals scheduled for magnetic resonance imaging during the study, those following a very low carbohydrate diet, individuals with food allergies or intolerances to study-related products, and those having ≤3 bowel movements per week on a regular basis. Further details regarding the inclusion and exclusion criteria can be found in Supplemental Information 1.

#### Sample size calculation

There is sparse literature on the luminal microbiota composition in the small intestine in healthy humans [19] and none on microbiota-induced lipid-derived metabolites inside the lumen of the human small intestine. Due to the proof-of-concept nature of this study, it was not possible to perform a sample size calculation. Instead, the sample size was based on feasibility and mean number of study participants in other human clinical trials that have included placement of a naso-intestinal catheter [6]. For this study, we aimed to include 16 participants. If catheter placement failed, ≤4 reserve participants could be included to ensure a total of 16 participants completing the study.

#### Recruitment and screening

Study participants were recruited from the surroundings of Wageningen through the database for potential participants of the division of Human Nutrition of Wageningen University and Wageningen Food & Biobased Research. Additional recruitment took place by posters and social media. An information booklet was provided to participants who expressed their interest in the study, and they were invited to an information meeting that was organized prior to the study start to inform them of the study aims and procedures. When participants were interested in participation, written informed consent was obtained first, after which screening took place. During screening, height and weight were measured to determine BMI, veins were assessed by a research nurse for cannula accessibility, and a questionnaire was completed, which contained questions regarding the inclusion and exclusion criteria.

### Study design

**FIGURE 1:**
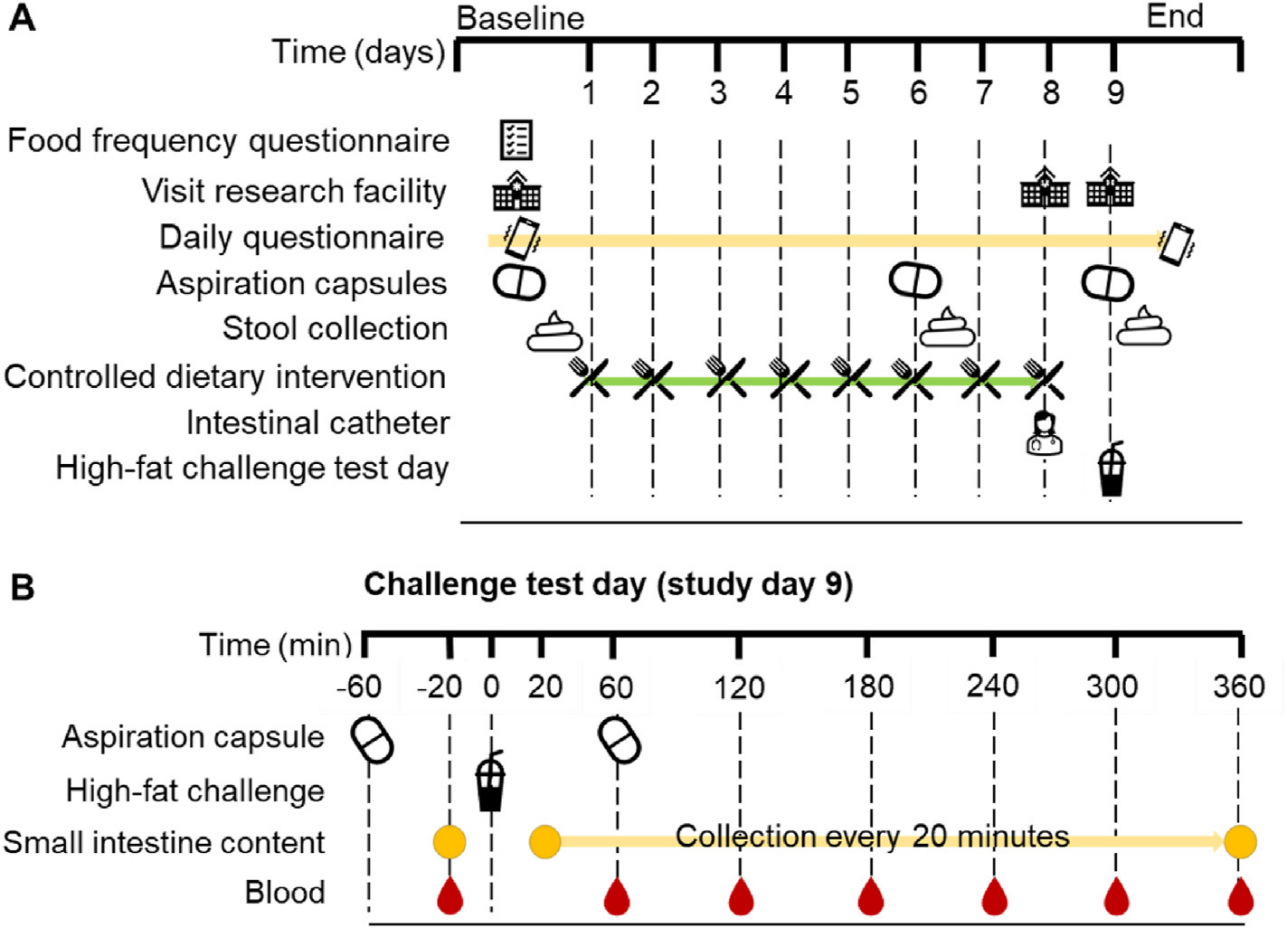
Design of the TAPIR proof-of-concept study. During the study (A), participants filled in questionnaires, ingested aspiration capsules on 3 different days, followed by stool sampling, and followed an 8-d plant-based mild-ketogenic diet. On day 8, a naso-intestinal catheter was positioned in the ileum, followed by a challenge test day. During this challenge test day (B) on study day 9, a high-fat shake was provided, 2 aspiration capsules were ingested, small intestinal content was collected every 20 min from the naso-intestinal catheter, and hourly blood samples were collected.

This study started with an 8-d preconditioning period with a plant-based mild-ketogenic controlled diet, followed by a challenge test day involving a high-fat shake on day 9. Aspirates from the small intestine were sampled via a naso-intestinal catheter before (T=0) and every 20 min ≤6 h postprandially on the day of the high-fat challenge. Blood samples were collected at baseline and every hour postprandially. Additionally, aspiration capsules (*n* = 2 per time point) were orally ingested before (day 0) and on day 6 of the preconditioning diet, as well as during the high-fat challenge (day 9, Figure 1). Participants retrieved the aspiration capsules from the feces and collected an aliquot of the fecal sample that contained the first capsule. Data management was conducted using CASTOR electronic data capture version 2024.1.1.1.

#### Daily questionnaires and reminders

At baseline only, the subjects completed a food frequency questionnaire to assess their habitual dietary intake [20,21]. During the full study period, starting after the first study visit, participants received short daily questionnaires to assess capsule intake and retrieval, compliance, stool pattern, and GI complaints via a modular research application on their smartphone, suitable for data sampling through push notifications (https://pocketq.net/). The questionnaires and reminders were created within the Qualtrics web-based platform version 2.5.0 (Qualtrics). Questionnaire push notifications were sent at 20:00 h. During the 8-d diet, participants received questions regarding their diet compliance, specifically whether they consumed anything other than the study meals. If so, they were asked to specify the items eaten, providing quantities and brands. Additionally, participants were asked to report whether all provided meals and snacks were fully consumed. If not, they were prompted to specify the items and the approximate leftover amounts. Furthermore, participants completed daily questions regarding GI complaints and stool patterns. Abdominal cramping, bloating, flatulence, and heartburn were reported on a 7-Points Likert scale. Stool pattern was reported as the number of bowel movements (stool frequency) and the consistency per bowel movement according to the Bristol Stool Form Scale (version Dutch May 2016, the Rome Foundation, Inc.). The days after capsule intake, participants received additional questions about the aspiration capsules, including whether they found the capsule(s), the number of capsules found, and the date and time of discovery. This information was used to calculate capsule transit times.

#### The 8-day controlled preconditioning dietary intervention

The research participants’ small intestines and their microbiota were preconditioned for 8 d with a plant-based mild-ketogenic diet. This preconditioning aimed to provide each individual with a more consistent background diet and to prime the microbiota of the small intestine for metabolizing lipids during the subsequent high-fat shake challenge [22]. The study by David et al. [13] suggests 5 d of switch in diet should be sufficient to see effects, particularly on microbiota composition and activity. However, for logistical reasons related to the timing of capsule intake and retrieval, catheter placement, and the challenge test, the diet duration was extended to 8 d. The diet was plant-based because animal products themselves may contain fermentation by-products, such as CLA [16]. Human cells lack the enzymatic machinery to convert LA into CLA. This conversion primarily occurs in ruminant animals, facilitated by specific gut microbiota [16,23]. The animal-free diet ensured that the potential production and detection of small intestinal microbiota-derived lipid metabolites, such as CLA, was not confounded by dietary CLA. For similar reasons, fermented products like soy yogurt and sauerkraut and other foods containing bacterial cultures were also excluded from the diet. To accurately compose this 8-d isocaloric diet, participants were classified into 1 of 3 energy groups based on the Schofield formula, utilizing anthropometric measurements (height and weight), age, gender, and general activity level (applied formulas in Supplemental Table 1). The diet contained 10%–20% of the total energy intake (EN%) from carbohydrates, 10%–20 EN% from protein, and 60%–70 EN% from fat (Table 1 for macronutrient breakdown). Notably, the diet was rich in LA, with an average daily intake of 15 EN%. Additionally, participants consumed plant sterols (custom-made spread by Flora Food Group, Supplemental Table 2) at the recommended maximal daily dose of 3 g, as recognized as safe by the Food and Drug Administration and the European Food Safety Authority [24]. Participants received meal boxes for breakfast, lunch, dinner, and snacks to prepare and consume at home. A 4-d meal plan was designed and repeated twice. On day 6, the diet was slightly different to accommodate the protocol for aspiration capsule intake, and on day 8, further adjustments were made to ease eating in combination with the placement of the naso-intestinal catheter. Meals included, for example, low carbohydrate sandwiches made from almond and linseed flour (Supplemental Information 2 for bread recipe), topped with peanut butter, hummus, vegan cheese, or plant-based meat (Supplemental Table 3 for meal planning details). Snack options included fruit, vegetable snacks, and healthy bars. Dinner selections featured dishes like zucchini spaghetti, pumpkin lasagna, broccoli- or cauliflower rice, all accompanied by vegetables, meat replacers, sauces, and a salad with a sunflower oil-rich dressing. High-fat desserts, such as peanut butter cheesecake, were also included. Participants were not restricted to specific mealtimes and had the flexibility to switch meals or products between eating occasions during the day. However, they were instructed to consume all meals allocated for the assigned day to maintain consistent caloric intake and EN% distribution throughout the 8-d diet.

**TABLE 1:** The dietary composition of the 8-d controlled dietary intervention and of the high-fat shake. The mean composition of all 8 d is provided for the 3 energy groups (size S, M, and L)

Nutrient	Meal size S (Day 1–8)	Meal size M (Day 1–8)	Meal size L (Day 1–8)	High-fat shake
Foodscore (g)	1203.0	1421.9	1654.1	375
Energy (kcal)	1839.7	2277.3	2634.0	885
Energy (kJ)	7611.3	9416.3	10892.3	3640
Total protein (g)	77.8	102.4	120.9	1.5
Total protein (EN%)	17.4	18.5	18.9	0.7
Plant-based protein (g)	77.8	102.4	120.9	1.5
Animal protein (g)	0.0	0.0	0.0	0
Total fat (g)	127.3	158.0	179.6	97.3
Total fat (EN%)	61.8	62.1	61.0	98.9
Total SFAs (g)	23.7	30.3	33.0	10.3
MUFAs cis (g)	44.2	55.2	62.4	28.4
PUFAss (g)	35.1	43.7	51.6	54.6
C22:6 n–3 cis (DHA) (g)	0.0	0.0	0.0	0.1
C18:2 n–6 cis (linoleic acid, LA) (g)	32.3	39.9	47.5	54.4
Total *trans* fatty acids (g)	0.1	0.1	0.1	0
C18:3 n–3 cis (ALA) (g)	2.4	3.2	3.5	0.1
C20:5 n–3 cis (EPA) (g)	0.0	0.0	0.0	0
Cholesterol (mg)	0.3	0.3	0.4	0
Total carbohydrates (g)	78.6	90.4	109.1	0.8
Total carbohydrates (EN%)	17.6	16.3	17.0	0.4
Total mono-and disaccharides (g)	46.3	51.1	65.9	0.5
Total polysaccharides (g)	29.8	36.7	40.7	0.3
Total fibers (g)	29.3	35.7	41.5	0.8
Total fibers (EN%)	3.1	3.0	3.1	0.2
Water (g)	711.7	812.9	957.5	274.2
Total alcohol (g)	0.0	0.0	0.0	0
Total alcohol (EN%)	0.0	0.0	0.0	0
Sodium (mg)	1040.3	1307.0	1412.3	94

Abbreviations: ALA, alpha-linolenic acid; EPA, eicosapentaenoic acid; DHA, docosahexaenoic acid; LA, linoleic acid.

#### Composition high-fat shake

On the challenge test day (day 9), research participants consumed a high-fat shake low in protein and carbohydrates to direct the microbial fermentation to dietary lipids. The high-fat shake consisted of 250 g of unsweetened almond milk (Albert Heijn supermarket), 95 g of sunflower oil (Flora Food Group), and 5 g of plant sterol (BASF SE, supplemental Table 2 for composition and mixing method). To enhance the palatability of the shake, 0.5 mL vanilla aroma (SC048029, International Flavors & Fragrances B.V.) was added to the 350 mL shake. This resulted in a high-fat shake containing 885 kcal, with 1.5 g of protein, 97.3 g of fat, and 0.8 g of carbohydrates (Table 1). The shake was freshly prepared on the morning of the challenge test day. The inclusion of a high-fat load aimed to facilitate the detection of microbial-derived lipid metabolites in the aspirate sampled from the small intestine via the catheter and the small intestine microbiome aspiration (SIMBA) capsules.

### The high-fat challenge test day

On day 8, the study participants visited the Gelderse Vallei hospital in Ede (the Netherlands) for catheter placement, which is described in more detail below in subchapter “Naso-intestinal catheter placement and progression.” On the morning of the challenge test day (day 9), the study participants returned to the hospital after fasting overnight. The placement of the catheter was checked using a single fluoroscopy image at the Radiology department to ensure it had progressed into the distal small intestine and that there were no coils in the stomach. The total length of catheter insertion through the nose was recorded. Following confirmation of successful positioning of the catheter, the test day proceeded at Wageningen University (Figure 1B). Participants ingested the first SIMBA capsule, marked with a blue marker (FD&C Blue #1, nonmedicinal inactive substance), 1 h before consuming the high-fat shake. Upon arrival at Wageningen University, a cannula was inserted into participants' forearms at the elbow crease for blood collection throughout the challenge test day. Initial sample collections included a baseline intestinal aspirate and blood sample. After baseline sampling, participants consumed the high-fat shake within 10 min (Table 1). One hour after consumption of the shake, the second SIMBA capsule (without the blue marker) was ingested with a glass of water. Intestinal aspirates were then collected every 20 min for the next 6 h, aiming for 2–3 mL aspirate per sample. These samples were collected using 5, 10 and 20 mL luer-lock syringes attached to the aspiration channel of the naso-intestinal catheter, gently drawing the syringe to collect the samples. First, the dead volume of the naso-intestinal catheter was collected (6.66 mL) in a spare 15 mL Eppendorf tube. Hereafter, it was attempted to collect additional small intestinal aspirate. In case additional sample could not be collected, the spare sample from the dead volume was used and stored. The aspirate was homogenized using a Pasteur pipette and aliquoted per 0.5 mL in prelabelled Eppendorf tubes on dry ice. At the end of each test day, all aliquots were stored at −80°C for future analyses.

During the 6-h postprandial period, also hourly blood samples were collected. At each blood drawing time point, the first 3 mL of blood was used to flush the cannula, after which 4 mL of blood was collected in a serum tube and a K_2_EDTA plasma tube. At baseline, 2 and 6 h after consumption of the high-fat shake, an additional 4 mL whole blood sample was collected in lithium heparin tubes for *ex vivo* experiments. A total of 89 mL of blood was collected during the challenge test day. The lithium heparin tubes were kept at room temperature for direct use in *ex vivo* experiments. Serum tubes were first left to clot at room temperature for 30 min and thereafter centrifuged at 3000 × *g* for 8 min, whereas K_2_EDTA plasma tubes were immediately cooled on ice water and centrifuged at 1200 × *g* for 10 min at 4°C within 1 h. To ensure an endotoxin-free environment during serum preprocessing, preparatory steps included washing hands, using endotoxin-free pipette tips, cleaning pipettes with 70% ethanol, and not wearing gloves. Aliquots of 0.5 mL plasma or 0.5 mL serum were transferred to prelabelled cryovials, immediately placed on dry ice, and subsequently stored at −80°C for future analyses.

Throughout the challenge test day, participants were instructed to drink 300 mL of water each hour to stay hydrated and to prevent the catheter and infusion from clotting, ensuring smooth flow. Participants were allowed to rest in chairs or beds in a study room at the research facility during and between measurements. At the end of the challenge test day, the cannula and the naso-intestinal catheter were removed, and participants were provided lunch. They also received materials for feces collection to continue until the capsules were retrieved. The hand-in of the final fecal sample and capsules during the last visit marked the end of study participation.

### Small intestine aspirate sampling

#### Catheter design

**FIGURE 2:**
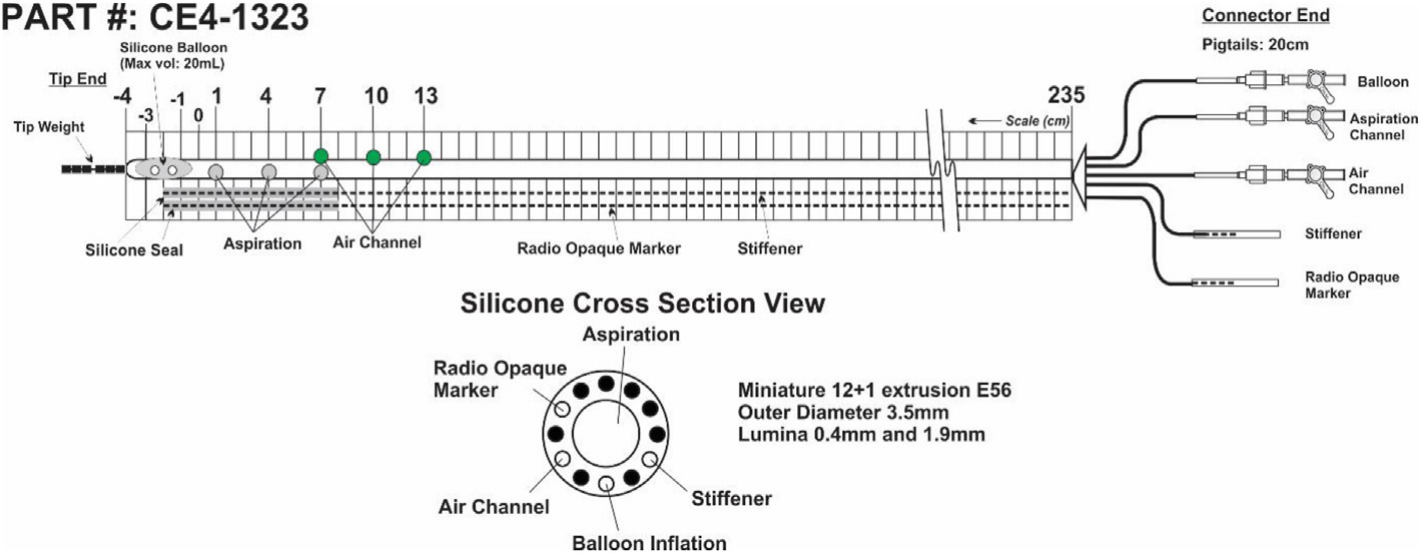
The design of the naso-intestinal catheter for sampling luminal fluid in the small intestine. The catheter had a total length of 255 cm, with a body length of 235 cm, a pigtail of 20 cm (the connector end), and an outer diameter of 3.5 mm. Multiple lumina were included in the design (see cross-section view), including an aspiration lumen with a diameter of 1.9 mm, a 0.4 mm lumen filled with a built-in stiffener, and a lumen for balloon inflation, with a radio-opaque marker for visualization, and for air inflation (in the case of aspiration difficulties). The 3 aspiration side holes were positioned at 1, 4, and 7 cm, and the air side holes were positioned at 7, 10, and 13 cm. At the tip end, a silicone balloon with a maximum volume of 20 mL was attached, and 2 tip weights (for catheter progression).

A custom-made naso-intestinal catheter (MUI Scientific) was used for sampling luminal fluid in the small intestine. A 255 cm long, silicone multichannel naso-intestinal catheter with an outer diameter of 3.5 mm was used (Figure 2). The body length was 235 cm long, and the pigtail length was 20 cm long. The aspiration channel had a diameter of 1.9 mm and contained 3 side holes at positions 1, 4, and 7 cm, with 3 cm interspacing between each side hole. Centimeter markings were included along the length of the catheter. One lumen was filled with a built-in 0.25 mm diameter stiffener to reduce the risk of coiling. A small (maximum volume 20 mL) deformable, inflatable balloon and 2 small weights were located at the distal tip end. The tip weights were encased at the distal tip to promote movement from the stomach into the duodenum. A radio-opaque marker was present along the full body of the catheter for visualization by fluoroscopy. The inflatable balloon was necessary for the progression of the catheter toward the distal small intestine using peristaltic movements. At the connector end, 1-way stopcocks were attached to open and close the lumen.

#### Naso-intestinal catheter placement and progression

**FIGURE 3:**
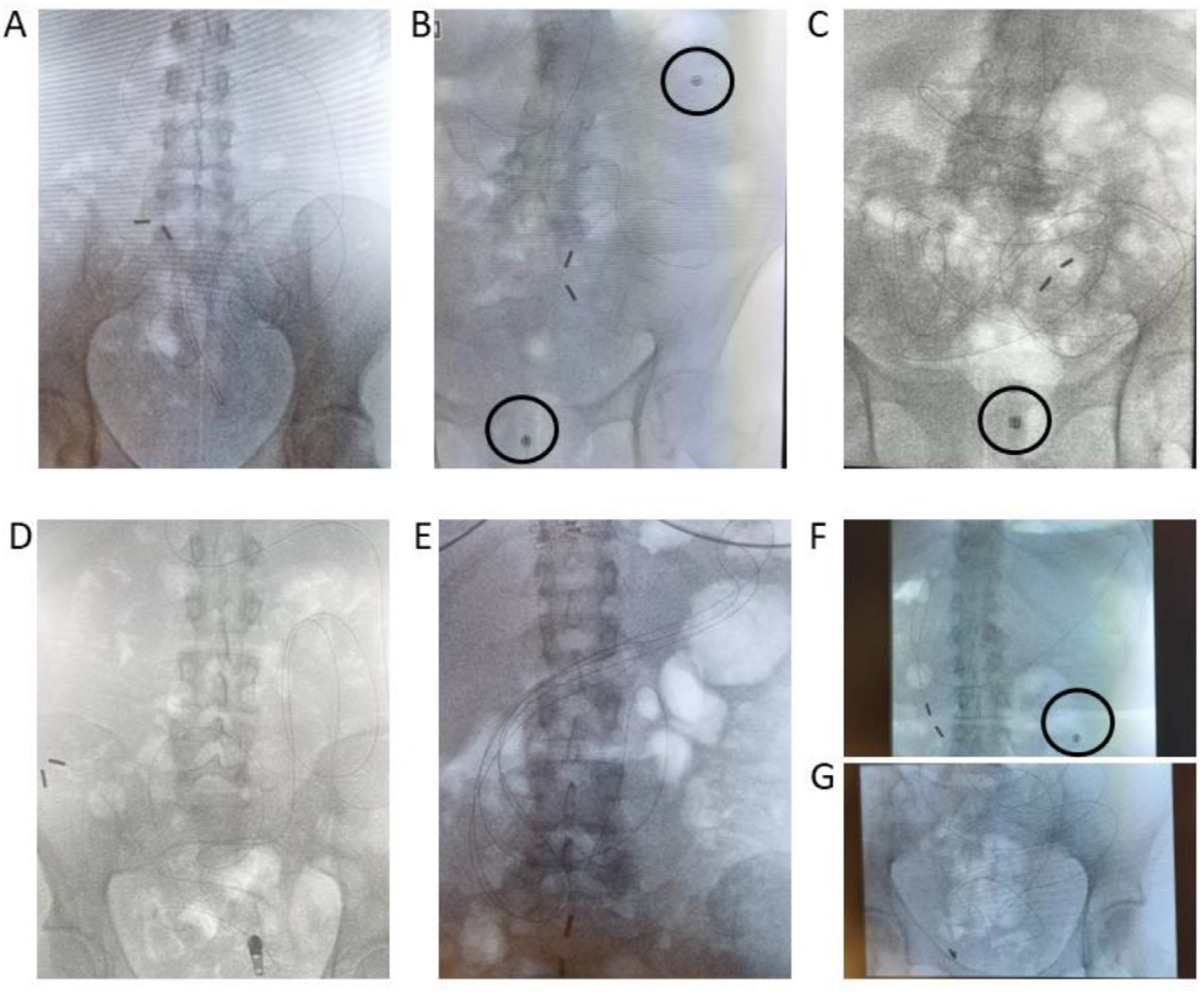
Examples of the visualization of the position of the naso-intestinal catheter and capsules (encircled) using fluoroscopy (day 8). The radio-opaque marker along the length of the catheter is visible at the distal end, where the 2 tip weights can be seen. (A) Naso-intestinal catheter inserted 168cm. (B) Naso-intestinal catheter 2 h after placement, inserted 130 cm, passed ligament of Treitz. Two SIMBA capsules ingested on day 6 are visible in the image. (C) The naso-intestinal catheter of the same participant in image B was inserted 200–220 cm, tip in the distal part of the ileum. SIMBA capsule is visible in the image. (D) Naso-intestinal catheter inserted 200–220 cm. Tip at the end of the ileum. pH was measured to confirm that the tip was still in the ileum. (E) Naso-intestinal catheter inserted 200–220 cm, with curling in the stomach. Tip of the naso-intestinal catheter was located in the jejunum. (F) Naso-intestinal catheter 2 h after placement, inserted 100 cm, tip located before ligament of Treitz. One SIMBA capsule is visible in the image. (G) Naso-intestinal catheter of the same participant in image F—inserted 205cm. Naso-intestinal catheter reached the distal part of the ileum. pH was measured to confirm that the tip was still in the ileum.

On study day 8, participants visited Hospital Gelderse Vallei in Ede (NL) in a fasted state (no eating and drinking after 22:00 h except for water to prevent gastro-esophageal reflux) for the insertion of the naso-intestinal catheter. The placement procedure of the naso-intestinal catheter was performed by a nurse under supervision of a gastroenterologist. Prior to placement of the catheter in the small intestine, a 270 cm, 0.025-inch single-use VisiGlide guidewire (Olympus America) was inserted inside the 1.9 mm lumen to further minimize the risk of coiling in the stomach. After local anesthesia of the nasal mucosa with Instillagel (lubricant containing lidocaine and chlorhexidine; Farco-Pharma GmbH), the catheter, smeared with Instillagel, including the guidewire, was introduced transnasally into the stomach. Subsequently, the tube tip was manually advanced into the proximal small intestine beyond the ligament of Treitz. The position was monitored throughout the placement procedure via intermittent fluoroscopic control for visualization of the radio-opaque marker (Figure 3) in the Radiology department. As per the gastroenterologist’s instructions, a fluoroscopic image was captured every 5 s (85kV and 320mA). Once positioned in the proximal small intestine, the guidewire was removed, and the balloon was inflated. The catheter was then allowed to migrate toward the ileum via intestinal peristalsis. After catheter placement, participants were provided with fluids and foods, such as a fruit shake and soup, which were part of the preconditioning diet, to promote bowel motility. Participants remained in the hospital until early afternoon under supervision for manual catheter insertion and balloon inflation. In short, catheter insertion proceeded manually at a maximum rate of 10 cm per hour, with the balloon continuously inflated with 5 mL of air. To maintain balloon inflation, any remaining air was removed every 30 min, followed by the reinsertion of 5 mL of air. If deemed necessary by the gastroenterologist or nurse, an additional check was conducted in the early afternoon in the Radiology department using fluoroscopy to monitor catheter progression and assess for stomach coiling. Participants were instructed to allow the tube to advance until a clearly marked point, 200 cm to a maximum of 220 cm, to potentially reach the mid- or distal ileum [6]. Once this point was reached, the balloon remained deflated, and the catheter was secured to the face with tape to prevent further movement. Participants received clear instructions and were provided with contact numbers (from medical personnel and researchers) for any questions or emergencies regarding the catheter. Eating, drinking, movement, and showering were possible during catheterization. Participants continued their preconditioning diet and remained fasted overnight (from 20:00 h onward), except for water, in preparation for measurements on the high-fat challenge test day.

#### Aspirate capsules

**FIGURE 4:**
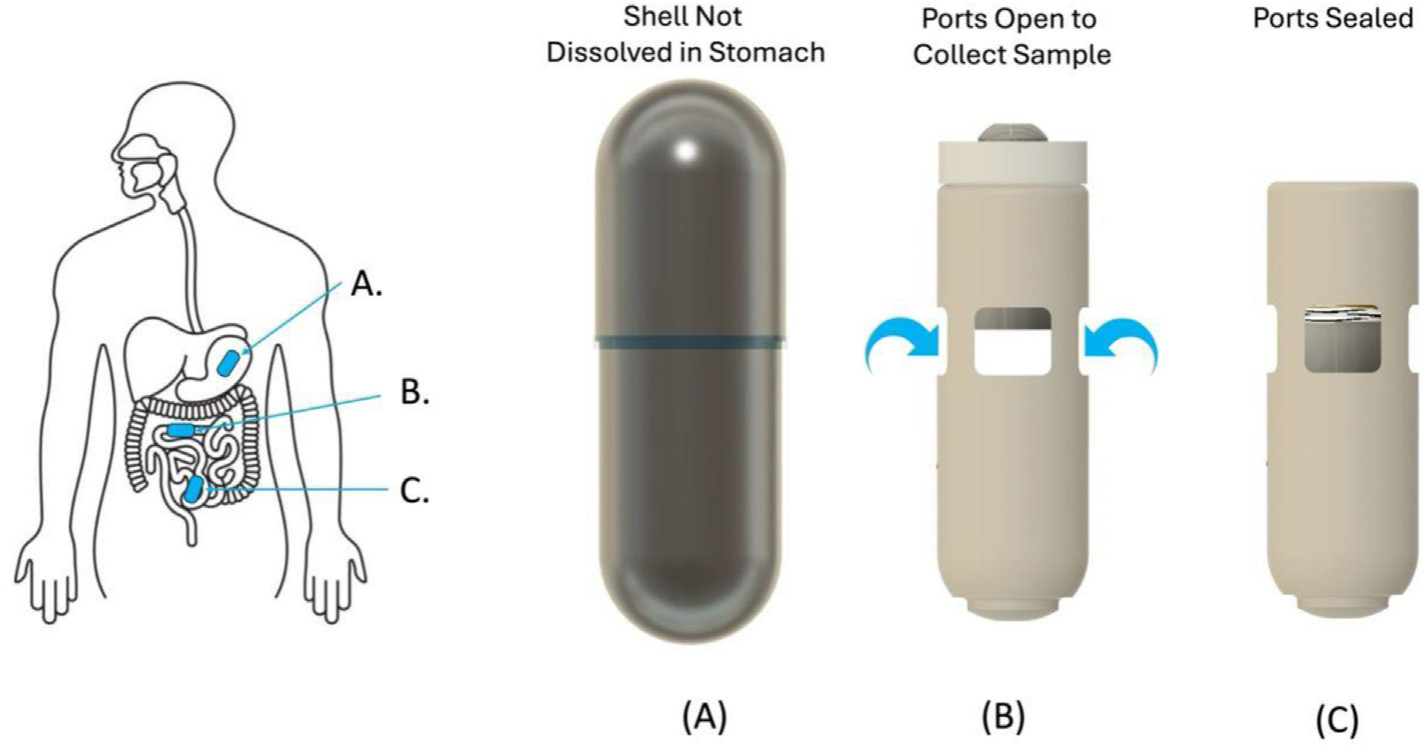
The configuration of the SIMBA capsule throughout the GI tract. The outer shell of the capsule remains intact in the stomach (A), and dissolves as it reaches the proximal small intestine, where it opens and collects a sample over time of the surrounding fluid (B). The capsule then closes before reaching the colon and remains sealed throughout the GI tract until expulsion and sample retrieval in the laboratory (C). Reproduced with permission from [26,28].

The SIMBA capsule (Nimble Science) was used for sampling luminal fluid along the small intestine in this study [[25], [26], [27], [28]]. The SIMBA capsule is a single-use, ingestible capsule [25 × 8 mm (L × D)] that allows for passive sampling, sealing, and preservation of luminal contents in the small intestine, Figure 4. The SIMBA capsule comprises a small plastic cylindrical container with fenestrated walls through which luminal fluid can passively wick. Contained within the device is a small spring-loaded silicone seal that is used to close the ports and prevent further ingress of fluid [26,28]. The entire apparatus is placed within a pH-dependent polymer coating, which dissolves at a neutral pH and thus passes through the acidic environment of the stomach intact. After dissolution of the external coating in the small intestine, the passive wicking is supported by hydrophilic fibers construct and contained within the device sampling chamber [28]. Integrated within the chamber is a food-grade bactericidal “stabilizer” substance, which deters further bacterial growth within the collected sample for the subsequent passage and collection time. Simultaneously with the onset of fluid sampling, the luminal fluid acts upon a second dissolvable substrate, which holds back the small spring-loaded seal. X-ray tracking in healthy volunteers and motility-challenged patients with irritable bowel symptoms (IBS) confirmed the dissolution of substrate takes place during the small intestine transit time before colon arrival [[26], [27], [28]]. The spring then seals the ports so that the device’s sampling chamber becomes impermeable to fluid, preventing contamination during colon transit [28]. The capsule, along with the captured intestinal fluid within it, is then excreted via feces collected by the study participant and returned to the laboratory for analysis.

#### Ingestion of capsules

At baseline (day 0, at the facility), after 5 full days of controlled preconditioning diet (day 6, at home), and at the challenge test day (day 9, at the facility), participants ingested 2 SIMBA capsules. After an overnight fast (only water was allowed after 22:00 h), participants drank 500 mL of water after waking up to rehydrate the GI tract. Thereafter, the 2 SIMBA capsules were ingested together with 250 mL water. The exact time of capsule consumption was registered. Capsule ingestion was followed by a wait of 2 h without eating and drinking (except water) to allow capsule clearance from the stomach, after which participants received a liquid breakfast shake of 200 calories. This breakfast shake contained blended strawberries (60 g), avocado (56 g), 90 g unsweetened almond milk (from Albert Heijn), 17.5 g soy protein powder (from Holland & Barrett), 3.5 g sweetener (liquid Natrena), and 123 g water. One serving of breakfast shake contained 350 mL and 200 calories. After this breakfast shake, participants again did not eat or drink for 1.5 h. On study day 9, intake of the 2 capsules was divided: the first capsule (blue marker) was taken 1 h before and the second capsule (without blue marker) 1 h after consumption of the challenge high-fat shake.

#### Retention of capsules and fecal sample collection

Participants received all materials to collect feces and to search for the capsules, namely labeled jars for collection of feces and collection of capsule(s), plastic nontransparent bags for storage, multiple cool packs, cooling bags, white buckets for fecal collection, a white tray to position the bucket in the toilet, and kits for searching the capsules (gloves, wooden searching sticks, trash bags). Urinating tubes (Urinelles, OmniMedical) were provided to female participants to separate urine from feces. Following the capsule consumption, participants collected all feces in the toilet using the materials until the capsules were retrieved. The fecal sample collected with the first capsule was stored in the home freezer, and capsule(s) were stored in the fridge. The capsules were processed in the laboratory within 6 d after ingestion of the capsule unless the capsule was not yet retrieved within that time span. When capsules were not expelled after 7 d, an abdominal X-ray was scheduled, as ordered by the gastroenterologist. Where the X-ray showed no capsules, it was assumed that capsules had passed and were lost in stool. When the X-ray showed that the capsules were still present in the intestine, regular observation in consultation with the gastroenterologist would have followed until the capsule had passed.

#### Retrieval of samples from the capsules

All tools necessary for sample extraction from the capsule were autoclaved at 121°C for 30 min before extraction began. First, the capsules were rinsed under running tap water to remove any feces on the surface. Next, each capsule was checked for intactness. The capsules were then placed on a removal tray and scrubbed with a cleaning brush for 10–15 s, followed by rinsing under running tap water for 30–60 s. This process was repeated until the capsule's surfaces were completely clean. The capsules and tray box were sprayed with 70% alcohol and transferred into a UV-sterilized biological safety cabinet. The capsules were then disinfected with 1% SDS, milli-Q water, and 70% alcohol and exposed to UV light inside the biological safety cabinet for 30 min. The plastic discs on top of the capsules were cut using scissors, and the fiber bundles were extracted from the twist cap collection chamber using straight tweezers. The fiber bundles were placed into labeled 2.0 mL screw cap tubes. Any remaining liquid in the capsules was transferred to the same tube using pipettes. To ensure all samples were retrieved, an additional 100 μL of sterile milli-Q water was added to each chamber, rinsed thoroughly, and then transferred to the same tubes. The capsules were weighed before and after removal of the sample to calculate the fiber weight. Finally, the tubes containing the liquid and fibers were stored at −80°C for future analyses.

### Planned data and biological sample analyses

An overview of the data and samples collected within the TAPIR proof-of-concept study, with the specific time points during the study at which they were collected, the types of biological matrixes involved, and the parameters that are planned to be analyzed, can be found in Table 2. The primary outcome measure of the study will be lipid-related metabolites (lipidomic analyses) in the catheter-collected small intestinal samples. As a secondary outcome, lipid-related metabolites will be analyzed in capsule aspirate, blood, and fecal samples. Other secondary outcomes will be the composition and/or activity of the microbiota in aspirate samples from the small intestine, in both catheter- and capsule-collected samples, and in fecal samples. Additionally, at selected time points, whole blood was used directly for *ex vivo* analyses, and subsequent supernatants were stored for future analyses of inflammatory markers.

**TABLE 2:** Overview of the data collected within the TAPIR proof-of-concept study

	Method or material	Main parameters	Study time point					
			Screening	Baseline	Daily during the study	Study day 6	Challenge test day 9	End
Dietary intake	Food frequency questionnaire	Habitual dietary intake		•				
	Digital short questionnaire via App	Compliance with the 8-d diet			•			
General health	Screening questionnaire	Health and lifestyle factors	•					
Anthropometrics	Digital weighing scale	Weight	•					
	Stadiometer	Height	•					
Stools and GI complaints	Digital short questionnaire via App	Self-reported GI complaints		•	•	•	•	
	Digital short questionnaire via App	Self-reported stool frequency		•	•	•	•	
	Bristol stool chart	Self-reported stool consistency		•	•	•	•	
	Digital short questionnaire via App	Capsule retention time, indicator for GI transit time		•	•	•	•	
Fecal microbiota composition and activity	Feces collection	Lipid metabolites, fecal microbiota composition		•		•	•	
Small intestinal microbiota composition and activity	Small intestinal content from capsules	Lipid metabolites, fasting small intestinal microbiota composition and activity		•		•	•	
	Small intestinal content from capsules	Lipid metabolites, postprandial small intestinal microbiota composition and activity					•	
	Small intestinal aspirates collected from the catheter	Lipid metabolites, fasting, and postprandial small intestinal microbiota composition and activity					•	
Baseline and postprandial parameters in blood	Blood sampling at baseline and after high-fat shake, followed by whole blood LPS stimulation assay	Inflammatory markers (cytokines, LPS, PGE2, HO1), triglycerides, lipid metabolites					•	
Evaluation of the use of capsules and catheter	Questionnaire	Self-reported study evaluation						•

### Statistical analyses

To evaluate our study protocol regarding capsule retrieval, biological sample collection, study compliance, stool pattern, and GI complaints, data analyses were performed using R version 4.3.3. *P* values < 0.05 were considered statistically significant. Linear mixed models using the “lmerTest” package in R were applied to examine differences in GI complaints, stool consistency, and stool frequency across study days and when grouped into prediet, during-diet, and postdiet periods. A linear mixed model was also applied to examine differences in capsule transit time across study days, using the mean transit time per day per participant. Differences between habitual dietary intake and dietary intake during the 8-d controlled diet were analyzed using paired T-tests, or Wilcoxon signed-rank tests, as appropriate based on the distribution of the dietary variables, with analyses conducted using the ‘rstatix’ package in R. The intrasubject and intersubject coefficients of variation (CoV%) for the capsule transit times were calculated using the formula CoV% = (SD/$\bar{x}$) × 100.

## Results

### Evaluation of participant enrollment

**FIGURE 5:**
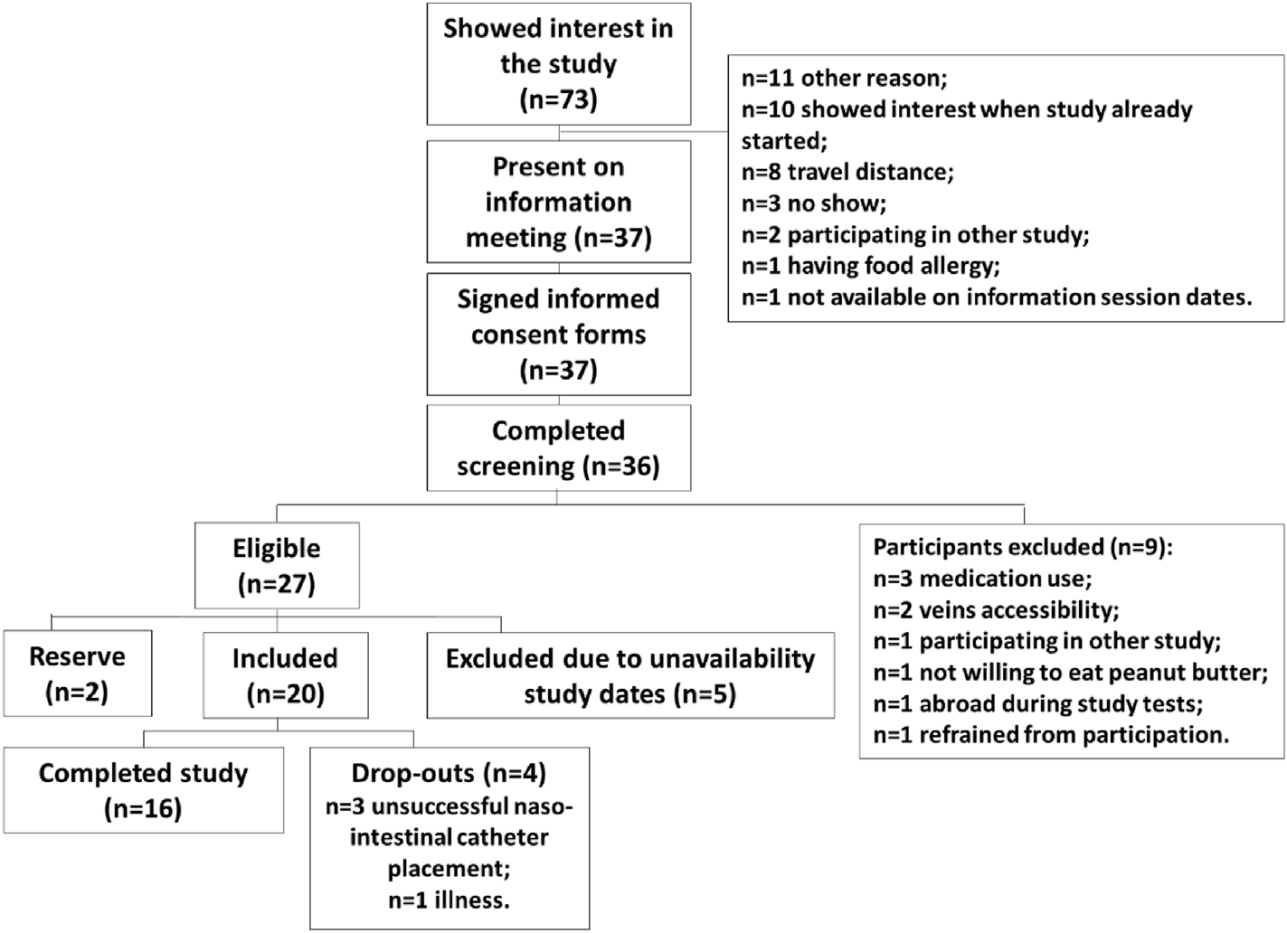
The study participant flow-chart of the TAPIR proof-of-concept study. In total, 37 participants signed informed consent forms, 20 participants were included in the study, of which 16 participants completed the study.

A total of 73 participants expressed interest in the study, with 37 providing written informed consent and 27 meeting the eligibility criteria based on the screening procedure (Figure 5). Ultimately, 20 participants were enrolled in the study based on availability, 10 females and 10 males, with a mean age of 48 y (range: 19–88 y) and a mean BMI of 24.3 kg/m^2^ (range: 19.5–30 kg/m^2^). Four participants dropped out of the study, with 3 failing to achieve successful placement of the catheter from the stomach to the small intestine and 1 withdrawing due to study-unrelated illness prior to catheter placement. The data collected from these participants until study termination will be included in the planned data analyses. Overall, 16 participants successfully completed the study, including the challenge test day.

### Evaluation of the 8-d controlled plant-based mild-ketogenic preconditioning diet

**FIGURE 6:**
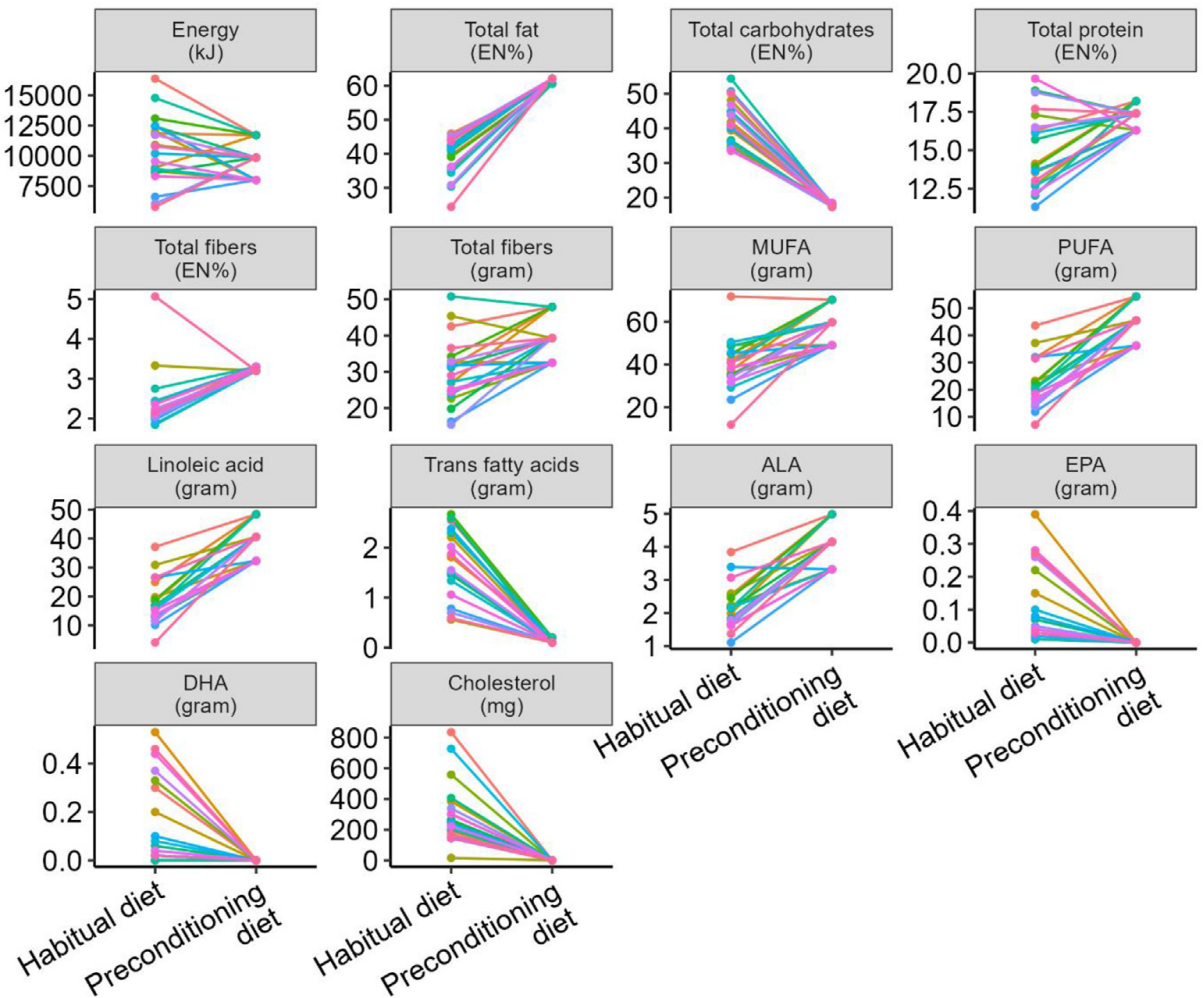
The energy, dietary lipids, and other macronutrients in the habitual diet and in the 8-d preconditioning plant-based mild-ketogenic diet. Note that for the isocaloric composition of the preconditioning diet, the study participants were divided into 3 energy groups based on gender, age, height and weight, and physical activity (Supplemental Information S2). Data from *n* = 20 individuals are included, as shown by the colored lines. ALA, alpha-linolenic acid; LA, linoleic acid.

The contrast between the habitual diet and the 8-d plant-based mild-ketogenic preconditioning diet was evaluated, including all 3 energy classification groups, to obtain insight into the changes in macronutrient composition (Figure 6 and Supplemental Table 4). The plant-based mild-ketogenic diet significantly increased the total fat EN% from 39.0 ± 5.72 to 61.7 ± 0.65, whereas significantly decreasing the total carbohydrates EN% from 42.0 ± 6.04 to 17.9 ± 0.49. The mean total dietary fiber increased from 30.0 ± 9.11 to 39.1 ± 6.04 g/d. Significant increases were also observed in the amount of MUFAs from 38.9 ± 12.0 to 58.6 ± 8.36 g/d, PUFAs from 22.1 ± 9.0 to 44.4 ± 7.15 g/d, and in particular LA from 18.0 ± 7.82 to 39.6 ± 6.36 g/d.

To assess dietary compliance to the plant-based mild-ketogenic diet, participants recorded daily whether they consumed any foods apart from what was provided during the 8-d preconditioning period. Overall compliance was high, with only 6 instances (3.9%) out of 153 recorded occasions where participants recorded consuming nonstudy foods, 4 of which were by the same participant who withdrew on day 8 due to illness. Participants also reported on the completeness of meal and snack consumption, with 92% (140 out of 153) of the meals and snacks being fully consumed. Noncompliance in finishing meals or snacks was reported to be due to reasons such as oversight, illness, or a strong dislike for specific foods.

### Evaluation of adverse events and GI complaints

**FIGURE 7:**
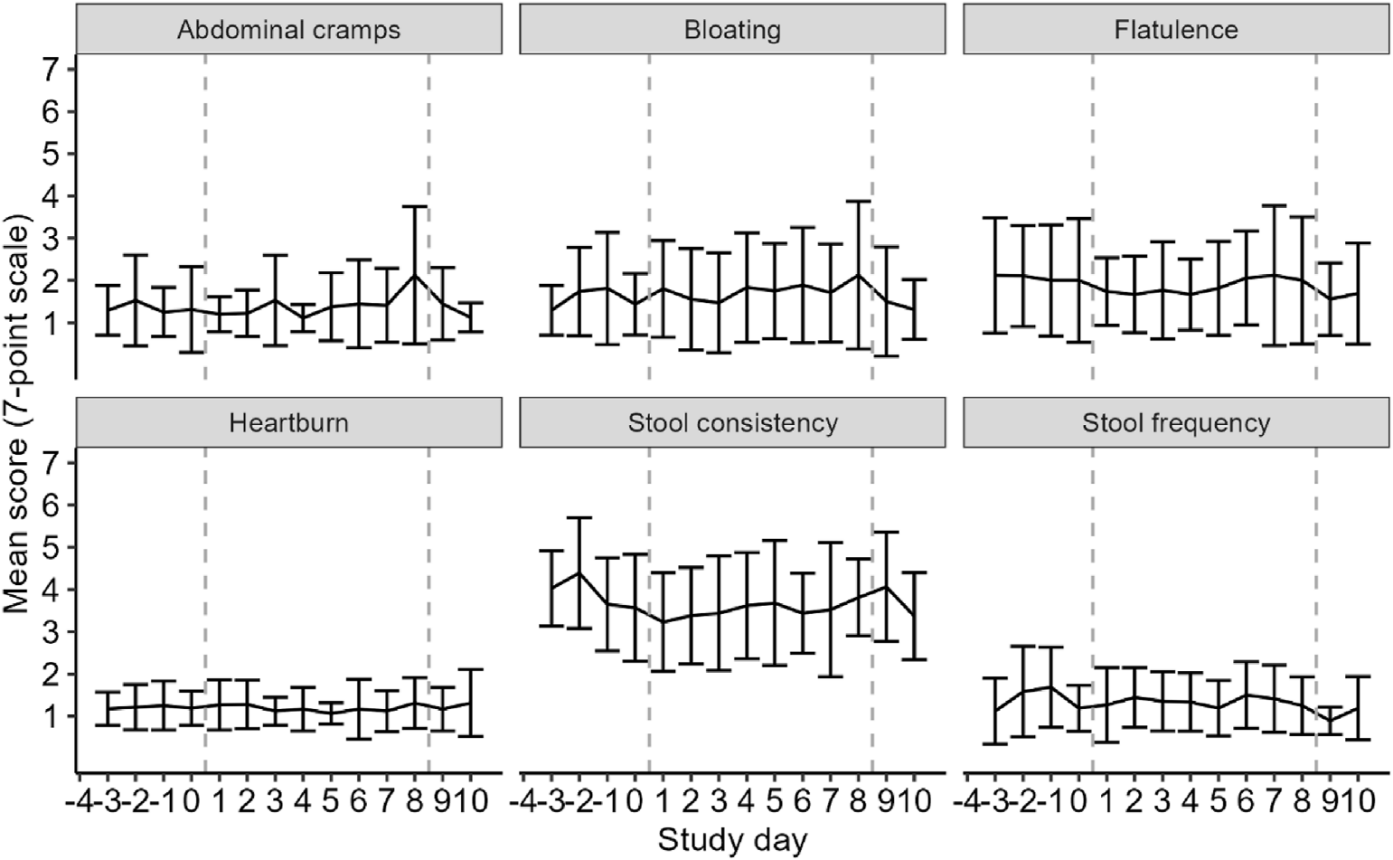
Self-reported GI complaints, stool frequency, and stool consistency during the study. Lines and error bars represent the mean values ± SD. Data from *n* = 7–20 individuals are included, depending on the number of missings, with a total of *n* = 237 observations per variable. The days in between the gray dashed lines correspond to the 8-d period during which the mild-ketogenic plant-based controlled diet was followed. Linear mixed models were applied to examine differences in GI complaints, stool consistency, and stool frequency across ∗ *P* values < 0.05.

During study execution, several adverse events (AEs) were reported, including discomfort and GI complaints, namely throat irritation (*n* = 5), abdominal cramping (*n* = 4), nausea (*n* = 2), vomiting (*n* = 2), headache (*n* = 2), flu-like symptoms (*n* = 1), and diarrhea (*n* = 1). Most of these AEs, such as throat irritation, nausea, headache, and flu-like symptoms, were likely related to the study procedures, such as naso-intestinal catheter placement or dietary intervention. Other AEs, including a cold (*n* = 1), food poisoning prior to starting the preconditioning diet (*n* = 1), and headache from a sports incident (*n* = 1), were considered unlikely to be related to the study procedures or products. During the study, participants reported daily GI complaints, stool consistency, and stool frequency (Figure 7). No significant differences in abdominal cramping, bloating, flatulence, heartburn, stool consistency, or stool frequency across the study days or between the periods before, during, and after the 8-d preconditioning diet were found (*P* values > 0.05).

### Evaluation of fecal and blood sample collection

The participants were instructed to collect feces from the stool from which the first capsule was retrieved. The total success rate for fecal sample collection was 94.8%. In cases where neither of the 2 capsules was retrieved, no stool sample was collected, which happened on 3 occasions (5.2%), of which 2 times on day 0 and 1 time on day 9. Blood samples were taken hourly during the challenge test day via a cannula. Cannula placement and blood draw were successful in all participants (*n* = 16, 100%).

### Evaluation of sampling small intestinal content using catheters and capsules

#### Catheter sampling

Participants were instructed to advance the naso-intestinal catheter to a length of 200–220 cm. Based on previous experiences and literature [6], the catheter tip was then expected to reach the mid- to the distal ileum. In 14 of the 16 participants, this was indeed the case. Although the catheter was advanced <200 cm in 6 participants, fluoroscopy images confirmed that the catheter still reached the expected mid- or distal part of the ileum in 4 of these cases, according to the gastroenterologist. For the remaining 2 of these 6 participants, partial curling of the catheter in the stomach was observed, leading to aspirate collection from the jejunum.

The average success rate of aspirate collection from the catheter, meaning withdrawal of ∼2 mL fresh intestinal aspirate or, in case this failed, the dead volume in the catheter, was 49% (Supplemental Table 5), though this rate varied significantly depending on the time point of collection. The highest success rate was found 120 min after ingestion of the high-fat shake (75%) and at the end of the test day (94%) when remnants were sampled from the catheter after its removal. The catheter had a dead volume of 6.66 mL, which was initially aimed to be discarded before sampling 2 mL of fresh aspirate for the analyses. In 66% of the cases, however, the aspirate volume was less than the dead volume, which was documented, and dead volume aspirate samples were stored for further analysis. The mean total volume of successfully collected, fresh or dead volume, aspirate samples was 1.7 ± 1.3 mL. Aspirate sampling lasted, on average, 9 ± 3 min. Collections exceeding 15 mi to aspirate any sample were considered unsuccessful, prompting a new attempt 5 min later at the next scheduled time point.

#### Capsules sampling

In total, *n* = 112 capsules were ingested by the participants in this study, of which *n* = 40 capsules on day 0, *n* = 40 capsules on day 6, and *n* = 32 capsules on day 9 (Supplemental Table 6). In total, *n* = 6 capsules were missed for day 0 ingestion, *n* = 3 capsules for day 6 ingestion, and *n* = 2 capsules for day 9 ingestion. This resulted in a successful retrieval of 85% of capsules on day 0, 94% on day 6, and 91% on day 9. The average successful retrieval of the total number of capsules was 89%. The average successful retrieval as per ingestion (i.e., a minimum of 1 capsule out of the 2 ingested on the same day was retrieved) was 94.7%. None of the capsules showed signs of damage after retrieval. Compliance with the capsule ingestion protocol was high. Two protocol deviations were documented, involving the timing of the second capsule ingestion on day 6, which occurred 20 and 50 min later than scheduled, respectively.

#### Capsule transit times

**FIGURE 8:**
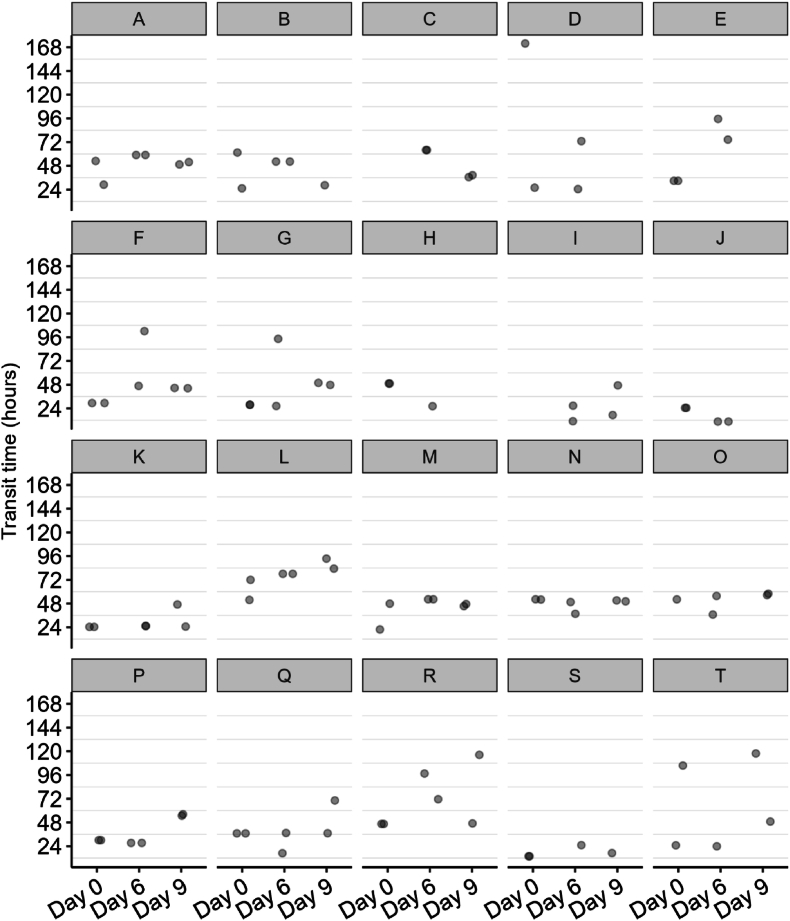
The transit times of the aspiration capsules per participant. The panels represent the data of the *n* = 20 individuals on day 0 and day 6, and for *n* = 16 individuals on day 9.

Next, the capsule transit times were evaluated. The median transit times were 32.8 h (range: 13.8–171.9 h) for day 0, 49.5 h (10.7–102.3 h) for day 6, and 48.3 h (17.0–117.8 h) for capsules taken on day 9 (Supplemental Figure 1). The capsule transit time was not significantly different between days (*P* = 0.31). The variability of transit times, both within and between participants, was also assessed. To calculate the intraindividual CoV% for transit times, first, the mean transit times for each participant were determined across days 0, 6, and 9. The mean intraindividual variability (CoV%) in transit time was 40.7% ± 20.1% (Figure 8 and Supplemental Table 7). The CoV% ranged from a minimum of 11.4% to a maximum of 93.7%. For interindividual variability CoV% calculations, the mean transit time per participant per ingestion day was used. The mean interindividual variability in capsule transit times was 42.5% ± 19.6% for day 0, 46.7% ± 23.0% for day 6, and 50.8% ± 19.9% for day 9.

#### Capsule sample weights

**FIGURE 9:**
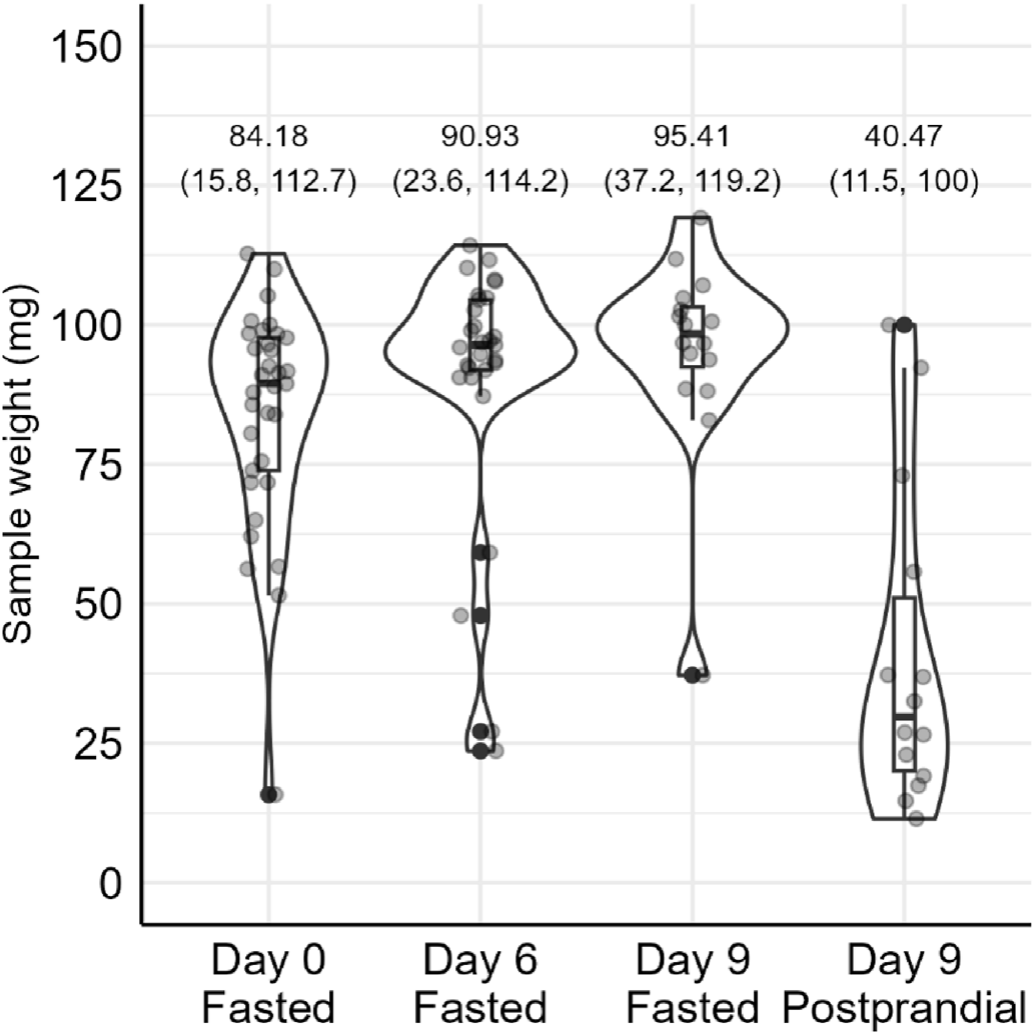
The intestinal sample weights extracted from the aspiration capsules. Day 9 data were split into capsules ingested in a fasted state and postprandial state. Day 0 fasted contains data of *n* = 34 capsules, day 6 fasted *n* = 37 capsules, day 9 fasted *n* = 16 capsules, and day 9 postprandial *n* = 14 capsules. Boxplots show the median, and the 25th and 75th percentiles. The values show the mean, minimum, and maximum retention time.

The mean sample weights extracted from the capsules ingested in the fasting state were highly similar across the different study days (Figure 9), with weights of 84.2 mg (range: 15.8–112.7 mg) at day 0, 90.9 mg (23.6–114.2 mg) at day 6, and 95.4 mg (range: 37.2–119.2 mg) at day 9. However, the sample weight of the capsules ingested in the postprandial state (1 h after shake consumption) on day 9 was substantially lower, namely 40.5 mg (range: 11.5–100 mg).

## Discussion

This TAPIR proof-of-concept intervention study was designed to confirm and explore the interaction between dietary lipids and the small intestinal microbiota. All participants consumed the same plant-based mild-ketogenic diet, which exposed the small intestinal microbiota to a high dietary lipid load, aiming at preparing the microbiota for the high-fat shake during the challenge test day.

Designing this preconditioning diet proved challenging due to its complexity and product requirements. The isocaloric controlled preconditioning diet yielded distinct dietary contrasts, with the mild-ketogenic diet resulting in a significantly increased total fat EN%, with significant increases in MUFAs, PUFAs, and especially LA, and a corresponding decrease in total carbohydrate EN%. Due to the complexity of the diet, omitting both animal-based products and fermented foods for their bacterial load and potential metabolites, it proved difficult to achieve sufficient levels of protein. To account for 10–20 EN% protein, which was similar to the habitual diet, it was decided to add vegan protein powder to some meal components, such as soup and sauce, which may have slightly affected taste and texture.

Transitioning to the plant-based diet notably increased daily dietary fiber intake from 30 to 39 grams. Drawing from existing literature, we speculate that this dietary change primarily influences colonic microbiota composition, as was previously measured in fecal samples [29], rather than directly impacting the small intestinal microbiota. Additionally, adopting a plant-based diet led to obvious reductions in total cholesterol, EPA, DHA, and *trans* fatty acids. Exploring a plant-based diet aligns with the pursuit of a healthy and sustainable lifestyle, making it an important factor to consider. Further investigation is needed to better understand the potential consequences of these dietary shifts on the small intestinal microbiota. However, we will be aware of potential nutrient-induced microbial changes other than the dietary lipids that were primarily studied, namely LA and plant sterols.

Transitioning to a high-fat diet has previously been linked to GI complaints, including diarrhea [30]. We, however, did not find any effect of the 8-d preconditioning diet on abdominal cramping, bloating, flatulence, heartburn, stool consistency, or stool frequency. This suggests a high level of tolerance among our participants, possibly due to the concurrent increase in dietary fiber content. Dietary fiber has known benefits for gut health, such as improving stool patterns, promoting beneficial microbes, and enhancing the production of short-chain fatty acids, potentially mitigating adverse reactions to other dietary changes [31]. A strength of our study was the high level of dietary compliance observed among the participants.

Studying the small intestinal microbiome has several technical challenges, mainly regarding sampling techniques. The strength of our study design is the inclusion of different small intestinal sampling techniques, specifically capsules, and catheters. We intend to compare the future analyses of microbiota composition and lipid metabolites in capsule samples against small intestinal samples collected with the catheter. An earlier study by Wang et al. [27] already showed that microbiota and metabolomic composition of the SIMBA capsules were similar to endoscopic aspirate samples. In our study, we now conducted intestinal sampling using both naso-intestinal catheters (considered the gold standard) and the novel, less invasive aspiration capsules. Catheter placement proved challenging, resulting in 3 dropouts (15% dropout due to catheter placement), which aligns with expectations based on literature underscoring the complexity and high risk of curling in the stomach associated with these types of flexible silicone catheters [6]. Furthermore, catheter sampling encountered issues such as incomplete dead volume discarding. Although we aimed at discarding the dead volume prior to sampling fresh aspirate, this appeared not to be feasible in about two-thirds of the sampling. This can result in microbial processes occurring within the tubing, which may influence the outcomes. Strategies for handling missing values in postprandial sampling of aspirates will need to be addressed in future statistical analyses. Further improving the catheter sampling methodology, especially regarding sampling time points and volume per time point, has significant potential to enhance the validity of the outcomes.

Reported adverse effects related to the naso-intestinal catheter, such as throat irritation, nausea, headache, and flu-like symptoms, are consistent with prior research using naso-intestinal catheters [6]. Capsule retrieval rates improved over the study duration, with a mean successful retrieval rate of 94.7% as per ingestion (i.e., a minimum of 1 capsule out of the 2 ingested on the same day was retrieved). Missing capsules were not detected on the fluoroscopy images. Capsule retrieval rates were comparable with previous experiences, where retrieval rates of 97.5% [26,28] or 93% [27] were reported. To further improve retrieval rates in future studies, options such as collecting entire fecal samples for laboratory examination (although more labor-intensive) or employing techniques to aid in capsule detection could be explored. We observed a nonsignificant increase in median capsule transit times throughout the intervention period, contrary to expectations based on the increased dietary fat and fiber content, which would typically result in shorter transit times [32,33]. The median capsule transit times of ∼32 to 50 h were slightly higher than capsule transit times expected from literature in healthy participants, where a total median transit time of 30 h [interquartile range (IQR: 23–48)] was reported [26,28]. The transit times found in our study were more than those in patients with IBS, where a median total gut transit time of 47 h (IQR: 24–54) was found [27]. Interestingly, there was a substantial variability in retention time of capsules ingested at the same time point by the same participant. The increase in an overall relatively high capsule transit time may partly be explained by participants withholding defecation until a convenient moment to search for capsules within the feces. Many participants perceived the process of searching for capsules as burdensome. To mitigate the risk of capsules not being excreted from the body, participants with any history of known or suspected stricture, mechanical gut obstruction, or oropharyngeal dysphagia were excluded from the study. Moreover, to reduce the risk of long capsule retention times, people who regularly have <3 bowel movements per week were also excluded from the study. Recent studies show that the risk that capsules, and specifically Pillcams, are not retrieved from the body ranges from 0.6% to 0.8% in patients without inflammatory bowel disease [34,35]. A technical review by the European Society of GI Endoscopy concluded that most capsule retentions are asymptomatic, with the capsule potentially remaining in the small bowel for months and being naturally expelled during follow-up [36]. Conservative observation is typically sufficient unless malignancy is suspected, with the risk of bowel obstruction realistically cited as close to 1:4000. The SIMBA capsule, however, with its shrinking size due to dissolving exterior coating and deploying spring, results in a final size smaller than 21 mm in length and ∼7 mm in diameter when passed with stool. Consequently, we expected that the risk of retention or obstruction should be substantially lower than existing literature rates, and indeed, in our study, there was no retention or obstruction of the capsules.

To enable better comparison between the contents sampled by aspiration capsules compared with catheter samples, measuring the pH could provide additional information about the sampling location, particularly because the capsules sample passively. Because no brown capsule samples were observed, the yellow-greenish colors already indicate sampling from the small intestine. The mean intestinal aspirate sample weights per capsule ingested in the fasted states (84–95 mg, range: 16–119 mg) were comparable to those reported previously (89 ± 27 mg, range: 15–130 mg, [26,28]). The capsules ingested postprandially exhibited lower mean intestinal aspirate sample weights (40.5 mg, range: 12–100 mg) than those ingested in the fasting state, possibly due to interference from the high-fat shake with the capsule coating or its impact on gastric emptying of the capsule or intestinal transit time [33,37,38]. Although the latter was not indicated by our data, because we did not find a higher within-subject variability of capsule transit times at day 9 (fasting and postprandial capsule) than both capsules ingested at day 0 (both fasted) and day 6 (both fasted). The postprandial capsule on day 9 was administered 1 h after consuming the high-fat shake to potentially increase the detection of dietary lipid metabolites, which might be missed by the fasted capsule if it moves ahead of the food bolus. The suitability of capsules for postprandial sampling in combination with various other shakes or foods warrants further investigation in future studies. In contrast, catheter sampling generally improved in the postprandial state compared with the fasting state, confirming its suitability for postprandial kinetics assessment. Therefore, combining samples obtained from the capsules (fasting) and catheters (postprandial) will result in a wealth of data that sheds new light on dietary lipid effects of the small intestinal microbiota.

If we can confirm that the microbiota in the small intestine interacts with dietary lipids in humans, this would suggest future possibilities to stimulate health via modulation of the small intestinal microbiota using lipids or other dietary approaches. The TAPIR proof-of-concept study will provide insight into specific features (e.g., specific bacteria, diversity, and functions such as specific biosynthesis pathways) and their role in explaining variability in postprandial lipid responses between individuals. Overall, this information has the potential to offer new insights for future mechanistic research in the dietary interaction with the small intestinal microbiota, also within the contexts of obesity, cardiovascular diseases, and other chronic noninfectious diseases.

## Author contributions

The authors’ responsibilities were as follows – LJD, MvT, GH, NW: designed research; KF, MvT, LJD, RD, CP: conducted research; MvT, KF, LJD: analyzed data; MvT, LJD, KF: wrote the paper; LJD, MvT: had primary responsibility for the final content; and all authors: read and approved the final manuscript.

## Data availability

Data described in the manuscript and statistical script will be made available upon request pending.

## Funding

This research was supported by the Dutch Ministry of Economic Affairs via a Public Private Partnership grant from the Top Consortium for Knowledge and Innovation Agri & Food, project LWV21.165/LSHM21077 “Happy Fat, Healthy Microbiome” (acronym TAPIR). The funding agency had no role in the collection, analysis, and interpretation of data, nor in the preparation, review, or approval of the manuscript. The following companies contributed to the funding of this project: AAK Netherlands B.V., Ausnutria B.V., Bioiberica S.A.U., Cargill R&D Centre BVBA, Fuji Europe Africa B.V., Flora Food Group, Nimble Science, and Novozymes Berlin GmbH (part of the Novonesis Group). Flora Food Group supplied the spread high in plant sterols, and the plant sterols in sunflower oil mixtures, and Nimble Science supplied the aspiration capsules used in the study, as described in the methods and materials section. The private partners had no role in the collection, analysis, and interpretation of data or preparation of the manuscript.

## Conflict of interest

The authors report no conflicts of interest.
