# Supplementary material for: How to Study the Effects of Dietary Lipids on the Small Intestinal Microbiome? Methodological Design and Evaluation of the Human HealThy fAt, haPpy mIcRobiome (TAPIR) Proof-of-Concept Study
Source: Curr Dev Nutr. 2025 Feb 5;9(3):104564. doi: 10.1016/j.cdnut.2025.104564 (PMC11908603; doi:10.1016/j.cdnut.2025.104564)
Supplement: multimedia component 1 [file mmc1.docx]

# Supplemental information 1. In- and exclusion criteria

**Inclusion criteria**

- Adult male or female
- BMI 18.5-30 kg/m2
- Suitable veins for insertion of cannula

**Exclusion criteria**

A potential research subject who meets any of the following criteria will be excluded from participation in this study:

- Having a history of medical or surgical events that may either put the subject at risk because of participation in the study, or influence the results of the study, including diabetes mellitus, dyslipidemia, a swallowing disorder, gastrointestinal or liver disease, irritable bowel syndrome, sleeping apnea, renal failure, cancer, nose/throat diseases, gastric bypass surgery, use of anticoagulants; as determined by the medical supervisor;
- Having a bleeding/coagulation disorder, including hemophilia, Von Willebrand disease, Bernard-Soulier, Glanzmann thrombasthenia or thrombocytopenia;
- Use of antibiotics within 3 months of starting the study or planned during the study;
- Use of any medications in the previous week that could substantially alter gastrointestinal motor function (e.g., opioids, prokinetics, anticholinergics, laxatives), or acidity (PPI, H2RA), as determined by medical supervisor; - Use of pro- and prebiotic supplements within 4 weeks of starting the study;
- Planning or scheduled to undergo magnetic resonance imaging (MRI) at any time during the course of the study (the SIMBA capsule is MR unsafe)
- Currently following a very low carbohydrate (ketogenic) diet;
- Having regularly less than 3 bowel movements per week (being constipated);
- Alcohol consumption >21 glasses a week (women) or >28 glasses a week (men);
- Pregnant, lactating or wishing to become pregnant in the period of the study (self-reported);
- Not willing to give up blood donation during the study;
- Food allergies or intolerances for products that we use in the study;
- Current smokers;
- Current users of soft and/or hard drugs;
- Participation in another clinical trial at the same time;
- Being an employee of the Food, Health & Consumer Research group of Wageningen Food & Biobased Research or Human Nutrition and Health Department of Wageningen University.

# Supplemental Table 1. Schofield formula

|  |  |  |  | Activity level | | |  |
| --- | --- | --- | --- | --- | --- | --- | --- |
| Female |  |  |  | 1 | 2 | 3 |  |
| Age | Weight in kg | Height in m | BMR | BMR*1.35 | BMR*1.55 | BMR*1.75 |  |
| 18-29 | 75 | 1.66 | 4.71 | 6.35 | 7.29 | 8.23 |  |
| 30-59 | 75 | 1.66 | 6.09 | 8.22 | 9.44 | 10.66 |  |
| ≥ 60 | 75 | 1.66 | 4.54 | 6.12 | 7.03 | 7.94 |  |
| Formula females:  18-29 yrs: (0.057*W)+(0.01184*H)+0.411  30-59 yrs: (0.034*W)+(0.006*H)+3.530  60+ yrs: (0.033*W)+(1.917*H)+0.074 | | | |  |  |  |  |
|  |  |  |  | Activity level | | |  |
| Male |  |  |  | 1 | 2 | 3 |  |
| Age | Weight in kg | Height in m | BMR | BMR*1.35 | BMR*1.55 | BMR*1.75 |  |
| 18-29 | 85 | 1.80 | 8.31 | 11.21 | 12.88 | 14.54 |  |
| 30-59 | 85 | 1.80 | 7.73 | 10.44 | 11.98 | 13.53 |  |
| ≥ 60 | 85 | 1.80 | 7.06 | 9.53 | 10.95 | 12.36 |  |

Formula males:
18-29 yrs: 0.063*W)-(0.00042*H)+2.953

30-59 yrs: (0.048*W)-(0.011*H)+3.670

60+ yrs: (0.038*W)+(4.068*H)-3.491

# Supplemental Table 2. Plant sterol-rich spread and oil

**Supplemental Table 2.** Nutritional information of the plant sterol-rich spread.

| \| Nutritional Information \| Per 100g \| \| --- \| --- \| \| Energy (kJ) \| 2219 \| \| Energy (kcal) \| 539 \| \| Fat (g) \| 60 \| \| of which \|  \| \| saturated fatty acids (g) \| 15 \| \| mono-unsaturated fatty acids (g) \| 17 \| \| poly-unsaturated fatty acids (g) \| 28 \| \| Carbohydrate (g) \| <0,5 \| \| of which \|  \| \| sugars (g) \| <0,5 \| \| Proteins (g) \| <0,5 \| \| Salt (g) \| 0 \| |  |
| --- | --- | --- | --- | --- | --- | --- | --- | --- | --- | --- | --- | --- | --- | --- | --- | --- | --- | --- | --- | --- | --- | --- | --- | --- | --- | --- | --- |

**Ingredient list**: vegetable oils (sunflower, rapeseed), plant sterol esters 26% (of which 15% plant sterols), water, coconut fat, emulsifier (lecithin, mono and diglycerides of fatty acids), acidifier (lactic acid) and natural flavoring.

## Plant sterol-rich oil

**Ingredient list:** 8.3g of sterol esters (Cholesterol <1%, Brassicasterol (max) 3%, Campesterol 14%, Campestanol 1%, Stigmasterol 1%, Betasitostanol 8%, Betasitosterol 71%), 91.7g of sunflower oil

Mixing method:

- Warm up the sunflower oil and sterols in the oven at 75°C until the sterols are completely fluid.
- Weigh the sunflower oil and the sterols.
- Put the sunflower oil on the hotplate with a magnetic stirrer at 75°C and 500 rpm.
- Slowly add the sterols to the oil.
- Stir for 1 hour.
- Put the mix in a scotch flask and store ambient.

# Supplemental information 2. Low-carb bread ingredients

**Ingredients for 9 breads:**

- 8500 gram almond flour
- 1500 gram lineseed flour
- 2000 gram gluten (20% of the flour)
- 170 gram salt (1.5% of the flour)
- 200 gram yeast
- 5200 gram water

# Supplemental Table 3. Meal planning

**Supplemental Table 3**. Meal planning of the 8-day mild ketogenic plant-based diet.

| Meal moment | Type | Day 1 | Day 2 | Day 3 | Day 4 | Day 5 | Day 6 | Day 7 | Day 8 |
| --- | --- | --- | --- | --- | --- | --- | --- | --- | --- |
| Breakfast | *Bread* | Low carb bread | Low carb bread | Low carb bread | Low carb bread | Low carb bread | Milkshake (as per protocol after fasting). | Low carb bread | Milkshake (as per protocol after fasting). |
|  | *Becel ProActiv* | Becel pro activ | Becel pro activ | Becel pro activ | Becel pro activ | Becel pro activ |  | Becel pro activ |  |
|  | *Bread topping 1* | plain hummus | peanut butter | hummus sun-dried tomato | peanut butter | plain hummus |  | hummus sun-dried tomato |  |
| Morning snack | *Fruit* | apple | orange | pear | mandarin (2 pieces) | apple | Not applicable. Ingestion of capsules in the morning. | pear | Not applicable. Placement of naso-intestinal catheter in the morning. |
| Fruit snack (only for L menu) |  | pear | apple | mandarin | apple | pear |  | orange |  |
|  | *Flapjack* | white chocolate raspberry | oat bars cocoa | smooth lemon | white chocolate raspberry | oat bars cocoa |  | white chocolate raspberry |  |
| Lunch | *Bread* | Low carb bread | Low carb bread | Low carb bread | Low carb bread | Low carb bread | Low carb bread | Low carb bread | Low carb bread |
|  | *Becel ProActiv* | Becel pro activ | Becel pro activ | Becel pro activ | Becel pro activ | Becel pro activ | Becel pro activ | Becel pro activ | Becel pro activ |
|  | *Bread topping 1* |  |  |  |  |  | pindakaas |  |  |
|  | *Bread topping 2* | vegetarian ham | vegetarian spreadable sausage | vegetarian grill sausage | vegetarian ham | vegetarian spreadable sausage | vegetarian ham | vegetarian grill sausage |  |
|  | *Bread topping 3* | vegan cheese | vegan cheese cumin | Vegan cheese tomato-oregano | vegan cheese | vegan cheese cumin | vegan kaas naturel | Vegan cheese tomato-oregano | Pumpkin-coconut soup |
|  |  |  |  |  |  |  | cup-a-soup Tomato |  |  |
| Afternoon snack | *Vegetables/fruit* | cherry tomatoes | snack cucumber | snack carrots | cherry tomatoes | snack cucumber | orange + apple | cherry tomatoes | unsweetened applesauce |
|  | *Flapjack* |  |  |  |  |  | smooth lemon |  | oat bars cocoa |
| Dinner | *starch substitute* | zucchini spaghetti | cauliflower rice | pumpkin lasagna | broccoli rice | zucchini spaghetti | cauliflower rice | pumpkin lasagna | broccoli rice |
|  | *vegetables* | macaroni vegetables | vegetables for fried rice | spinach | ratatouille vegetables | macaroni vegetables | vegetables for fried rice | spinach | ratatouille vegetables |
|  | *sauce* | Tomato sauce | satay sauce | Tomato-chili sauce | soy sauce | Tomato sauce | satay sauce | Tomato-chili sauce | soy sauce |
|  | *meat replacer* | Vegetarian Swedish meatballs | plant-based chicken pieces | vegan minced meat pieces | Vegan spicy tofu strips" | Vegetarian Swedish meatballs | plant-based chicken pieces | vegan minced meat pieces | Vegan spicy tofu strips" |
| Salad (as part of the dinner) | *raw vegetables* | salad with pickled onions | paprika salad | salad with radish | salad with cucumber | salad with pickled onions | paprika salad | salad with radish | salad with cucumber |
|  | *Dressing* | oil-vinegar-mustard | oil-vinegar-mustard | oil-vinegar-mustard | oil-vinegar-mustard | oil-vinegar-mustard | oil-vinegar-mustard | oil-vinegar-mustard | oil-vinegar-mustard |
|  | *Topping* | Pumpkin seeds | almonds | pine nuts | peanuts | Pumpkin seeds | almonds | pine nuts | peanuts |
| Dessert (as part of the dinner) | *Dessert* | brownie | peanut butter cheesecake | chocolate mouse | brownie | peanut butter cheesecake | brownie | peanut butter cheesecake | chocolate mouse |

# Supplemental Table 4. Habitual diet vs controlled diet composition

**Supplemental Table 4.** The differences in energy, macronutrients, and dietary lipids in the habitual diet compared to the 8-day preconditioning controlled diet.

| Nutrient | Habitual diet | Preconditioning diet | P-value |
| --- | --- | --- | --- |
| Energy (kJ) | 10405 ± 2785 | 9662 ± 1464 | 0.15 |
| Total fat (EN%) | 39.0 ± 5.72 | 61.7 ± 0.65 | 1.91E-06* |
| Total carbohydrates (EN%) | 42.0 ± 6.04 | 17.9 ± 0.49 | 1.91E-06* |
| Total protein (EN%) | 14.9 ± 2.59 | 17.2 ± 0.76 | 0.002* |
| Total fibers (EN%) | 2.36 ± 0.72 | 3.26 ± 0.05 | 2.69E-05* |
| Total fibers (gram) | 30.0 ± 9.11 | 39.1 ± 6.04 | 8.10E-05* |
| MUFA (gram) | 38.9 ± 12.0 | 58.6 ± 8.36 | 6.37E-07* |
| PUFA (gram) | 22.1 ± 9 | 44.4 ± 7.15 | 1.46E-09* |
| Linoleic acid (gram) | 18.0 ± 7.82 | 39.6 ± 6.36 | 3.93E-10* |
| Trans fatty acids (gram) | 1.66 ± 0.68 | 0.12 ± 0.04 | 1.91E-06* |
| ALA (gram) | 2.17 ± 0.67 | 4.07 ± 0.65 | 1.68E-09* |
| EPA (gram) | 0.12 ± 0.12 | 0 ± 0 | 9.44E-05* |
| DHA (gram) | 0.15 ± 0.18 | 0 ± 0 | 0.0003* |
| Cholesterol (mg) | 305.6 ± 200.2 | 0.29 ± 0.08 | 1.91E-06* |

Data are shown as mean ± SD for n=20 individuals. Groups were compared with paired t-tests (total gram of fibers, MUFA, PUFA, linoleic acid, ALA, cholesterol), or with Wilcoxon t-tests (energy, total fat EN%, total carbohydrates EN%, total protein EN%, total fibers EN%, trans fatty acids, EPA and DHA). ALA, alpha-linolenic acid; DHA, docosahexaenoic acid; EPA, eicosapentaenoic acid; LA, linoleic acid; MUFA, mono-unsaturated fatty acid; PUFA, poly-unsaturated fatty acids.

# Supplemental Table 5. Overview aspirate samples

**Supplemental Table 5**. Overview of successful aspirate samples collection (n=16). Successful aspiration also encompasses samples in which less than the ideal amount of 2-3mL was collected, as well as dead volume samples.

| Timepoint (min) | Percentage of successful collection of aspirates |
| --- | --- |
| 0 | 38% |
| 20 | 63% |
| 40 | 38% |
| 60 | 56% |
| 80 | 38% |
| 100 | 50% |
| 120 | 75% |
| 140 | 50% |
| 160 | 38% |
| 180 | 31% |
| 200 | 63% |
| 220 | 38% |
| 240 | 38% |
| 260 | 38% |
| 280 | 50% |
| 300 | 50% |
| 320 | 50% |
| 340 | 38% |
| 360 | 94% |

# Supplemental Table 6. Aspiration capsule transit times

**Supplemental table 6.** Success rates of capsule retrieval from the feces.

|  | Day 0 | Day 6 | Day 9 | Total |
| --- | --- | --- | --- | --- |
| Number of ingested capsules | 40 | 40 | 32 | 112 |
| 'Lost' capsules | 15% (n=6) | 8%  (n=3) | 6% (n=2) | 10% (n=11) |
| Successful retrieval of capsules (total number of capsules) | 85% (n=34 capsules) | 92% (n=37 capsules) | 94% (n=30) | 89% (n=101) |
| Successful retrieval of capsules per ingestion (minimal 1 capsule out of the two ingested per participant) | 90% (n=18 from 20 ingestions) | 100% (n=20 out of 20 ingestions) | 94.1% (n=16 out of 17 ingestions) | 94.7% (n=54 out of 57 ingestions) |

# Supplemental Figure 1. Aspiration capsule transit times


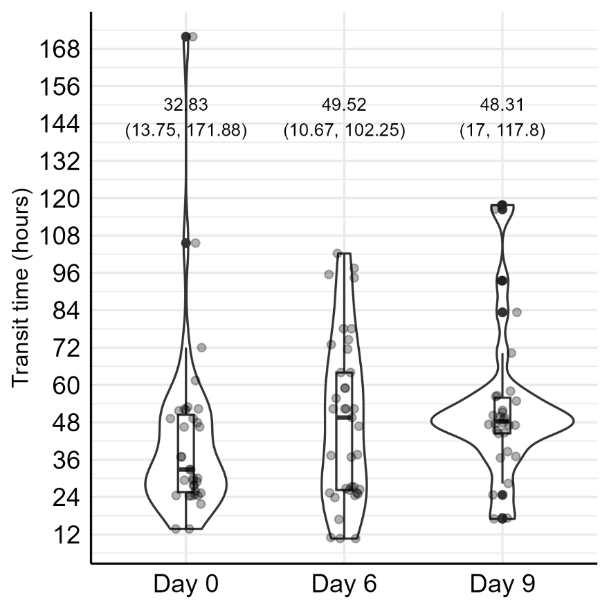


**Supplemental Figure 1**. The transit times of the aspiration capsules. Each day includes data on two capsules for each participant. Day 0 fasted contains data of n=34 capsules, day 6 n=29 capsules, day 9 n=30 capsules. Boxplots show the median, and the 25th and 75th percentiles. The values show the median, minimum, and maximum retention time.

# Supplemental Table 7. Intra-individual variability in capsule transit times

**Supplemental Table 7**. Intra-individual variability in capsule transit times. All available capsule retention times (day 0, day 6, day 9) were included in the analyses. The data is linked to the panels presented in Figure 8.

| Panel | Intra-individual coefficient of variation (%) | Retention time capsules  (Hours, mean ± SD) |
| --- | --- | --- |
| A | 22.15 | 50.19 ± 11.12 |
| B | 36.66 | 43.95 ± 16.11 |
| C | 30.07 | 50.79 ± 15.27 |
| D | 93.69 | 73.82 ± 69.16 |
| E | 53.15 | 58.93 ± 31.32 |
| F | 54.60 | 49.47 ± 27.01 |
| G | 57.26 | 45.64 ± 26.13 |
| H | 31.90 | 41.56 ± 13.26 |
| I | 62.08 | 25.58 ± 15.88 |
| J | 45.42 | 17.58 ± 7.99 |
| K | 31.92 | 28.52 ± 9.1 |
| L | 18.36 | 76.12 ± 13.97 |
| M | 25.83 | 44.54 ± 11.51 |
| N | 11.44 | 48.83 ± 5.59 |
| O | 16.72 | 51.87 ± 8.67 |
| P | 37.09 | 37.62 ± 13.95 |
| Q | 43.91 | 39.22 ± 17.22 |
| R | 42.48 | 70.91 ± 30.13 |
| S | 30.56 | 17.38 ± 5.31 |
| T | 69.68 | - 1. ± 44.72 |
